# Identification of ligand binding sites in intrinsically disordered proteins with a differential binding score

**DOI:** 10.1038/s41598-021-00869-4

**Published:** 2021-11-19

**Authors:** Qiao-Hong Chen, V. V. Krishnan

**Affiliations:** 1 Department of Chemistry and Biochemistry, California State University Fresno, Fresno, CA 93740 USA; 2 Department of Pathology and Molecular Medicine, University of California Davis, Davis, CA 95616 USA

**Keywords:** Biophysical chemistry, Chemical biology, Structure-based drug design

## Abstract

Screening ligands directly binding to an ensemble of intrinsically disordered proteins (IDP) to discover potential hits or leads for new drugs is an emerging but challenging area as IDPs lack well-defined and ordered 3D-protein structures. To explore a new IDP-based rational drug discovery strategy, a differential binding score (DIBS) is defined. The basis of DIBS is to quantitatively determine the binding preference of a ligand to an ensemble of conformations specified by IDP versus such preferences to an ensemble of random coil conformations of the same protein. Ensemble docking procedures performed on repeated sampling of conformations, and the results tested for statistical significance determine the preferential ligand binding sites of the IDP. The results of this approach closely reproduce the experimental data from recent literature on the binding of the ligand epigallocatechin gallate (EGCG) to the intrinsically disordered *N*-terminal domain of the tumor suppressor p53. Combining established approaches in developing a new method to screen ligands against IDPs could be valuable as a screening tool for IDP-based drug discovery.

## Introduction

Using computational algorithms to dock small molecules to proteins with ordered 3D structures to screen potential ligands is essential in a drug discovery process. The in silico screening is routinely applied in the early stages in rational structure-based approaches in selecting a ranked set of priority leads for experimental validation^1–3^. Docking methods initially utilized rigid ligands against static protein structures, as in influenza virus^4^ or HIV-1 protease^5^. As proteins sample multiple conformational sub-states, the ensemble docking approach notably advanced the virtual screening approaches closer to experimental conditions^6,7^. Ensemble docking for drug discovery pipeline has become one of the critical elements in the arsenal to develop potential drug molecules, as exemplified by the recent applications to find drug targets for Covid-19 infection^6,8–10^.

Intrinsically disordered proteins (IDPs) emerge as promising druggable targets due to their functional association with various diseases^11^. Screening ligands directly binding to an ensemble of IDPs to discover potential hits or leads for new drugs is exceptionally appealing because the ligands may directly block the undesired biological interactions mediated by the IDPs. However, to take advantage of the well-established structure-based rational drug design strategies, these methods need to be repurposed for IDPs that lack well-defined or ordered 3D- structures.

It is advantageous to access well-defined, folded, and experimentally generated three-dimensional structures for a ligand docking protocol. However, the inherent complexity of the IDP's conformational flexibility introduces an increased complexity to ensemble docking procedures. Therefore, the methods to identify binding sites of potential drug molecules to IDPs are current challenges in structure-based rational drug discovery. For the intrinsically disordered proteins when represented as an ensemble of structures for a docking protocol, two critical factors need to be distinguished concerning other conventional studies involving an ensemble of structured proteins. The differential binding score (DIBS), the difference in probability between two sets of ensemble docking protocols, is proposed to address these two factors. First, given an ensemble of conformations posed by an IDP, a potential ligand will bind to a selective subset of IDPs at a given moment but not necessarily in the same configuration because the subset of proteins with similar affinity may not have the same binding definitions to the ligand. The dynamic interconversion between the conformations of the protein is responsible for the differential binding events, leading to a probabilistic nature of protein–ligand interaction. A binding score defined must reflect how many such events of receptor-ligand interactions occur from a reasonably large set of binding events sampled. The second factor investigates the specificity of the IDP-ligand binding events by testing if similar events would occur if an ensemble of random coil structures represented the protein of interest. In combination, the procedure can then address that a particular ligand prefers a conformational subset posed by the IDP more specifically than random distribution, and the portions of the protein responsible for the interaction can be deduced.

In the method proposed herein, the two criteria mentioned above are measured, first by repeated sampling of ligand interaction with an ensemble of IDP conformations generated by molecular dynamics simulations and an ensemble of random coil conformations. From a large number of conformations (1000) each, a subset of populations (100 structures each) is randomly sampled for the ensemble docking protocol. Each sampled population is subjected to an ensemble docking for 24 independent docking routines to a total of 2400 runs for each sub-population. The same protocol is repeated three times separately for the IDP and random coil ensembles on a subset of conformations selected each time randomly. A differential binding score (DIBS) is defined based on the estimated binding affinity of each docking run and the number of times a particular amino acid is involved in the binding event. Secondly, linear modeling of the triplicate data was performed between the binding scores to identify residues that show significant differences leading to the identification of preferred binding sites on the IDP ensemble.

To demonstrate the utility of the approach, the method was tested on the binding of the ligand epigallocatechin gallate (EGCG) to the *N*-terminal domain of the tumor suppressor p53 (p53-NTD, an IDP)^12^. Zhao and Blayney et al. have revealed, based on their Surface Plasmon Resonance (SPR) and Nuclear Magnetic Resonance (NMR) studies, EGCG preferentially and directly interacts with the p53-NTD in a highly dynamic fashion with multiple binding interfaces. The DIBS supports these observations and the heterogeneous complex formation of EGCG with disordered proteins^12,13^. Furthermore, the differential binding score (DIBS) for the p53-NTD binding to EGCG shows a close agreement with experimental chemical shift perturbation data reported by Zhao and Blayney et al.^12^.

## Results

### Profiles of the probability scores of the random coil and IDP ensembles of p53-NTD

The ensemble of conformations of the MD simulations to represent the IDP populations and the corresponding random coil simulations are generated. Supporting information (Fig. S1) summarizes the characterization of the ensembles. The MD simulation analysis (Fig S1a, root-mean-squared deviation (RMSD) vs. time and Fig.S1b root-mean-squared fluctuation (RMSF) vs. AA #) shows a highly dynamic ensemble of conformations of p53-NTD. The backbone RMSD of the MD simulations shows a rapid increase with high dynamic fluctuations during the initial portion of simulations (Fig. S1a). Though the RMSD values do not continue to increase with increasing simulation time, the IDP ensemble continues to be highly dynamic throughout the simulation period. The IDP ensemble exhibits a distinct distribution of radius of gyration (Rg) as compared with the random coil ensemble (Fig. S1c). In addition, RMSD values of the IDP ensemble have a similar distribution as the Rg values (Fig. S1d). The random coil ensemble shows a broad distribution with a mean value of Rg (~ 22 Å). The IDP ensemble shows two populations with a major population centered close to the random coil population (~ 25 Å) and a second ensemble of conformation at a slightly higher Rg value (~ 40 Å). A principal component analysis (PCA) of ensembles (Fig. S1e,f) shows that the IDP ensemble better discriminated along with the first component, in comparison with the random coil ensemble (45.5% vs. 20.6%) and with similar discrimination along with the second component (~ 10%).

**Figure 1:**
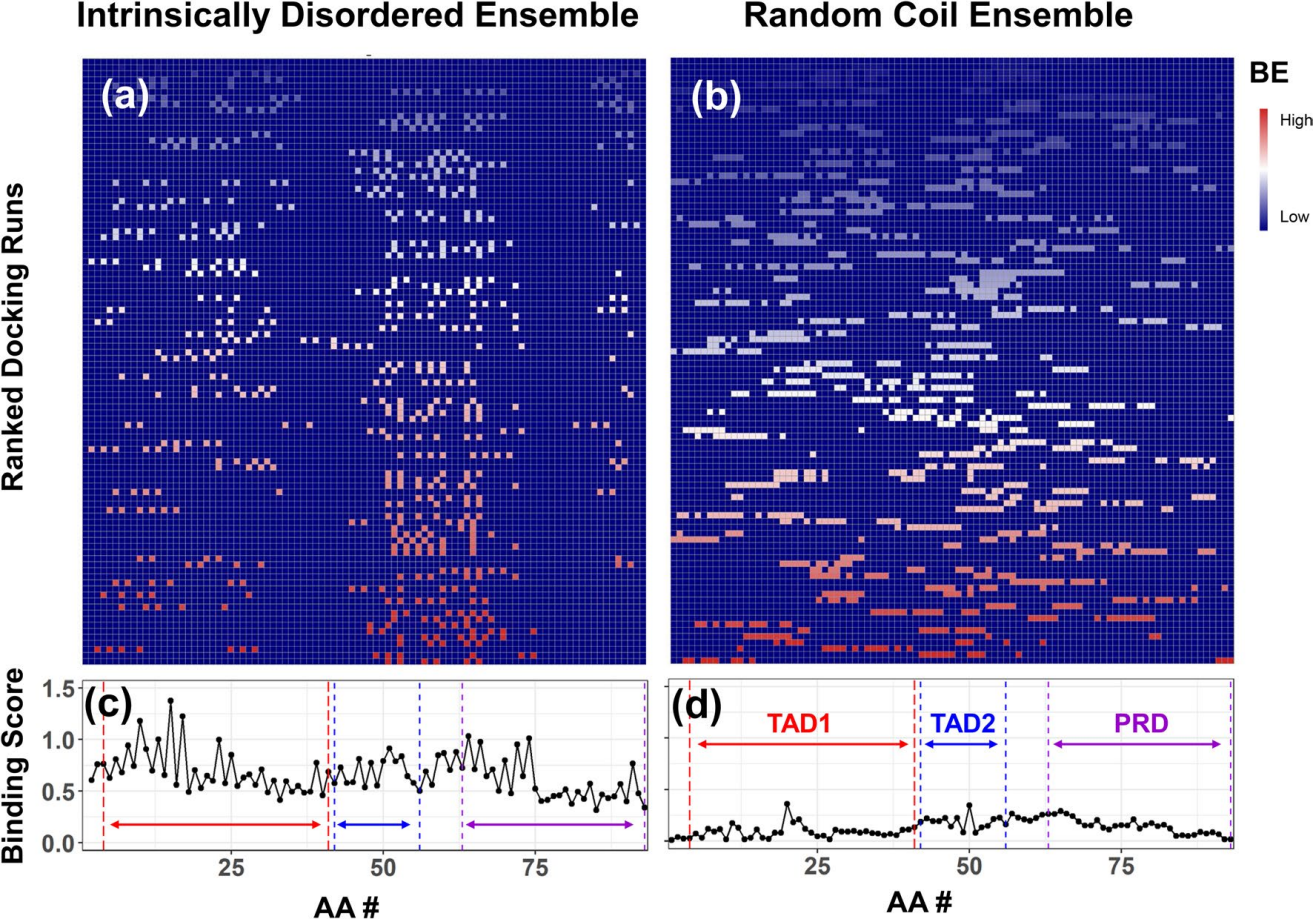
Docking runs and the binding score of p53-NTD interaction with EGCG. Top panels **(a,b)**: the intensity plot shows the binding energy (BE) from low (blue) to high (red) for each docking run (rows) for each of the amino acids that come in contact for that particular run. A representative set of top 100 binding runs is shown for ensembles with the MD generated **(a)** and the random coil **(b)**. Lower panels **(c,d)**: plot of the binding score (Eq. 1) plotted as a function amino acid residue position of p53-NTD. The transcriptional activation domains (TAD1 and TAD2) and the proline-rich domain (PRD) are marked.

The affinity of the EGCG is hypothesized to be differential between the ensembles of conformations. During each binding run, the interaction of the ligand and the receptor will lead to a measure of the binding constant (in kcal/mol), and the amino acid residues encounter the ligand. A representative example of the first 100 docking run results is shown in Fig. 1. For the first 100 runs (top rank at the bottom), the intensity of each square is the binding energy of that particular run (rows) plotted as a function of the amino acid residue sequence number (Fig. 1a,b). The top-ranked run to the p53-NTD as the IDP ensemble (Fig. 1a, bottom-most row) has a binding constant of 8.30 kcal/mol, and a dissociation constant of 817.24 nM, with the following interacting residues: Q52, W53, Y55, E56, D57, P58, G59, P60, P64, K65, M66, P67, E68, A69, and A70 with EGCG. For the p53-NTD random coil ensemble, the top-ranked run yields a binding constant of 8.34 kcal/mol and a dissociation constant of 766.47 nM with the ligand interacting residues E3, P4, S6, D7, P8, E11, P12, L14, W91, P92, and L93. Even though the binding constants of EGCG to the IDP or random coil are similar (8.30 kcal/mol vs. 8.34 kcal/mol), the essential residues that interact in these two runs are not the same. In a single docking run (as performed in the traditional approach), these results would imply that both the protein structures have the same affinity to the ligand, which does not reflect the reality when the receptor is represented by an ensemble of conformations. Suppose the ligand prefers a specific region of the receptor; the conformational selection should then be expected with higher consistency for the IDP ensemble vs. the random coil ensemble. The differential binding score (DIBS) is designed to amplify these measures systematically. In the intensity plots of Fig. 1, the IDP ensemble (Fig. 1a) and the receptor-ligand interactions tend to be more specific to certain protein regions, while in the random coil (Fig. 1b) is dispersed and less specific.

Cumulatively, the ensemble docking runs representing the p53-NTD binding to EGCG are given by the binding scores for the IDP ensemble (Fig. 1c) and random coil ensemble (Fig. 1d). When the ligand preferentially selects a specific region of the receptor, the binding score over the multiple runs increases, similar to how the signal averaging increases the signal-to-noise ratio. Accordingly, the binding score for the IDP ensemble (Fig. 1c) selectively increases for specific amino acid locations involved in the binding process. In contrast, the ones that have fewer interactions average out to with a low binding score. Figure 1d shows the regions defined as the transcription factor activation domain 1 (TAD1, P4-D41), transcription factor activation domain 2 (TAD2, D42-E56), and the proline-rich domain (PRD, A63-L93). Within the ensemble docking runs (Fig. 1), the IDP ensemble of the p53-NTD has preferred sites in both the TAD1 and PRD domains with higher binding scores than the same regions in the random coil ensemble.

### The differential binding scores (DIBS) defined between IDP vs. random coil ensembles

**Figure 2:**
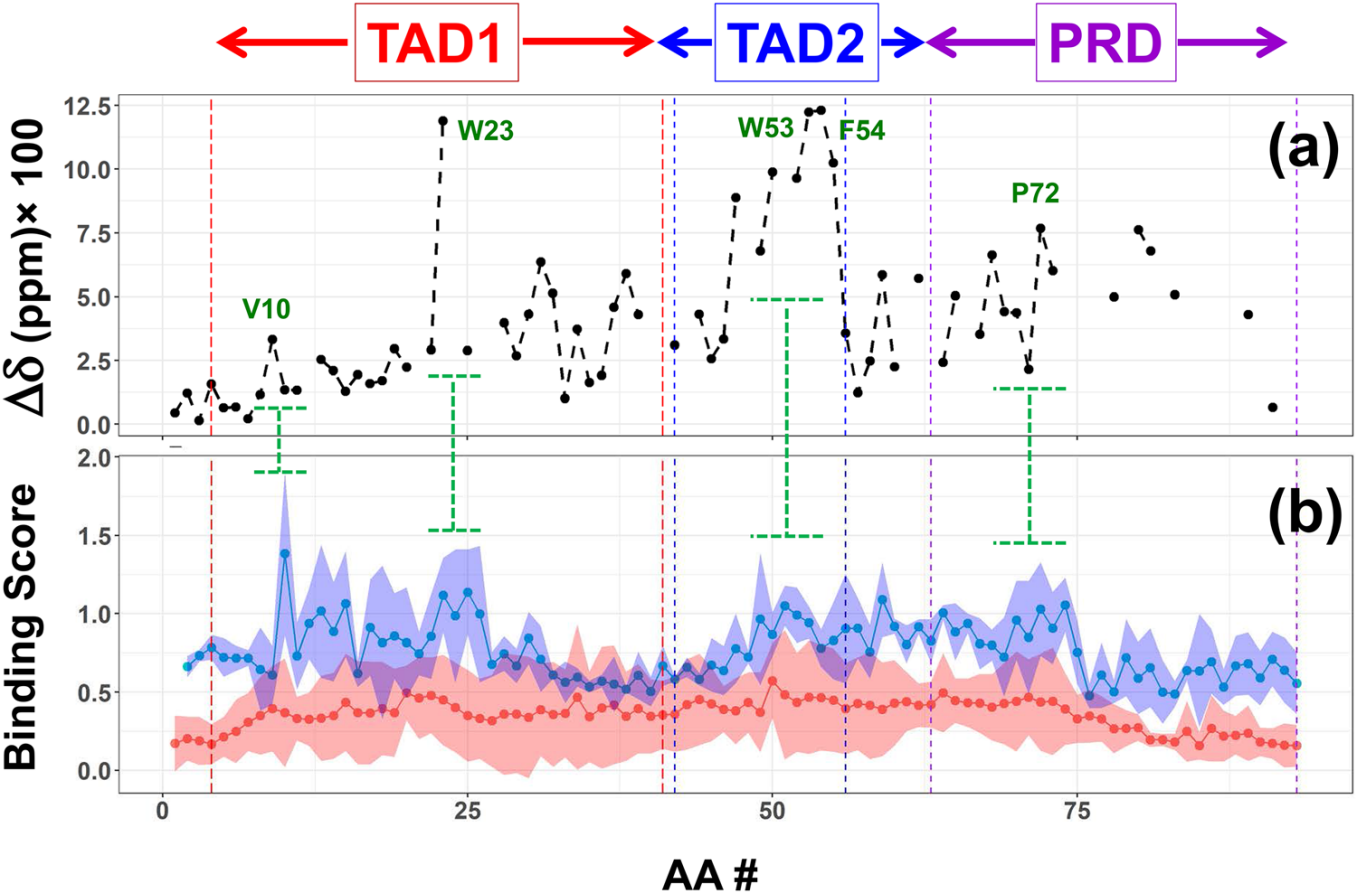
Comparison of the experimental chemical shift perturbation data with the binding scores. **(a)** Cumulative change in the chemical shifts of p53-NTD interaction with EGCG defined by weighted Euclidian distance of ^15^N and ^13^CO chemical shifts of the particular residue. Blanks correspond to missing experimental data. **(b)** A plot of the predicted binding scores of the IDP ensemble docking of p53-NTD with EGCG (blue) and the random coil ensemble of p53-NTD docking with EGCG (red). The average values of the three independently repeated measures are given by points, and the ribbons show the corresponding standard deviations of the measurements. The transcriptional activation domains (TAD1 and TAD2) and the proline-rich domain (PRD) are marked on the top. Amino acid positions that show a close match between the experimental **(a)** and the predicted **(b)** values are shown by the vertical lines (green).

The chemical shift perturbation data from Zhao and Blayney et al.^12^ is plotted in Fig. 2a. Instead of the changes in individual chemical shifts of either ^15^ N or ^13^C (Fig. S2), the effective chemical shift change that combines the ^15^ N and ^13^C chemical shifts is plotted (ratio of p53-NTD EGCG = 1:4). The original chemical shift data and the prediction of the intrinsic disorder of p53-NTD are provided in the supplemental information (Figure S2a). The binding score of many of the amino acid residues match with the experimental data, and these include V10, W23, V31, L32, S37, Q38, A39 (in TAD1), residues P47, I50, E51, W53, F54, and T55 (in TAD2) and M66 and P72 (in PRD). W23 shows one of the most considerable chemical shift changes, while V10 shows the largest change in the binding score. Residues S33-L35 show chemical shift changes (^13^CO chemical shifts, Fig. S2b) do not show notable changes visually, while residues, P27 and N30 are past the cutoff for significance (vide infra).

The ensemble docking was performed on a set of conformations selected for the IDP, and random coil conformations show a notable difference in the profiles. Are these binding events are statistically significant? To measure the differential effect, the procedure (Fig. 1) was repeated three times for each of the IDP and random coil ensembles of p53-NTD binding to EGCG. Figure 2b shows the summary of the results. The average variation in the binding score as a function of the amino acid position of p53-NTD is plotted for the IDP ensembles (blue lines) and the random coil ensemble (red line) of Fig. 2b. The blue and the red ribbon represent the standard deviation of the binding score over the three ensemble docking runs. The binding score plot shows several selective residues of preferential interaction between the p53-NTD with EGCG in the IDP ensemble compared to the random coil ensemble. In particular, residues in the TAD1 (V10, P13, S15, L23, and L25), few residues in the smaller TAD2 (D49, and E51), and several residues in the PRD (G59, P64, M66, A70, P72, and A74) have preferential interactions with EGCG. In contrast, such interactions with the random coil ensembles are absent. The amino acid residues that tend to interact with EGCG also happen to be clustered at the N-terminal part of the TAD1, interface between the TAD2 and PRD, and the N-terminal domain of PRD.

Considering that p53-NTD is an IDP (Fig. S2a), even at a spectrometer frequency of 800 MHz, it is impossible to identify all the NMR chemical shifts assignments due to an overlap of resonances or line broadening effects induced by the protein motion (Fig. S2). Besides, the lack of a proton in the proline residues sometimes leads to the absence of ^15^N chemical shift information (Fig. S2b). Because of these reasons, the chemical shift assignments of some of the residues may not be available. Therefore a residue-by-residue comparison between the two data sets (chemical shift perturbation vs. binding score) is incomplete.

### Identification of the residues of differential significance due to EGCG binding

**Figure 3:**
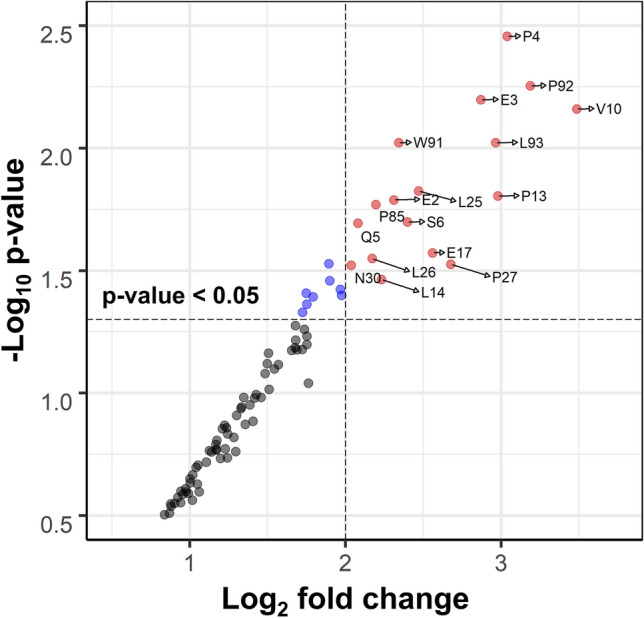
Volcano plot for the differential binding score (DIBS). The plot of the DIBS (fold change in log2) and the p-value on the differential preference of EGCG over the ensemble of IDPs than the random coil ensemble of the p53-NTD. The significance is defined with fold change > 2 and p-value < 0.05, marked by vertical and horizontal dashed lines. The black symbols represent residues that do not differentiate between IDP and random coil ensembles, blue symbols fulfill the p-value cutoff (< 0.05) and not the fold change (< 2.0) and the red symbols fulfill both the conditions for significance (fc > 2.0 and p-value < 0.05).

Repeated sampling of the subset of conformations from the IDP and random coil ensembles allows testing if these measured effects have any statistical significance, such as testing for the null hypothesis. For example, a particular amino acid residue of p53-NTD is considered significant if the binding score in that residue in the IDP ensemble is higher than that of the random coil ensemble with a p-value of the statistical test less than 0.05. These measures are generally shown in a plot of the fold-change vs. p-value, known as the volcano plot. A volcano plot for the statistical measure between the random coil ensemble and the IDP ensemble for repeated measures (three times each) is shown in Fig. 3. For a DIBS (fold change, (log2) > 2 and p-value < 0.05) are marked by the vertical and horizontal dashed lines, respectively (Fig. 3). In addition, the amino acids that pass the significance threshold are marked. Residues found at the upper right corner of the plots are the most significant, having the largest fold change with a low p-value. The opposite side of a typical volcano plot is missing in Fig. 3. No residues show higher binding affinity to a random coil ensemble than the IDP ensemble (negative fold change). Figure 3 indicates that P4, V10, and P92 significantly influence the preferential binding of EGCG. Other residues of varying importance are also noted in Fig. 3. The blue dots show the residues with p-values < 0.05, but the fold changes are not > 2.0, while the black symbols are residues that do not play a role in the differential binding. The residues that show a significant differential binding tend to be clustered more in the TAD1 and PRD than in the TAD2, also seen in Fig. 2.

Statistical analysis of the results from the ensemble docking runs to identify potential interactions between p53-NTD and EGCG is an essential element of the approach. This requirement confirms that EGCG has a higher affinity for IDP conformations than the sampled random coil conformations.

## Discussion

A differential scoring approach distinguishes the ensemble docking results between intrinsically disordered vs. the random coil states of a receptor. When the method is applied to assess the binding of p53-NTD with EGCG, in addition to providing a close match with the NMR-based chemical shift perturbation studies (literature results), other residues are also identified. Typically, docking studies are performed only with a specific receptor or with an ensemble representation. The value of the method relies on the fact that the differential binding score (DIBS) establishes a means to identify binding sites selected explicitly by the ligand on an intrinsically disordered ensemble over a random coil ensemble. Multiple sampling of conformations and a systematic statistical test suggest that this method could serve as a screening tool to identify potential ligands that bind to IDPs.

The study has limitations from the fundamental assumptions upon the ensemble docking concept that considers that the ligand selects a protein conformation based on the induced fit model^14^ and conformational selection and population fit models^15–17^. The presumption of success of this method depends on the hypothesis that the conformations generated and used for repeated sampling for the ensemble docking runs are sensitive enough for the EGCG to bind to the IDP than the random coil ensemble preferentially. However, in a broader sense, considering the number of conformations sampled by a protein typical of the size of p53-NTD in either the IDP or random coil ensemble is much larger than the size of the conformations used in the study. Thus, through the selection of a more extensive set of conformers might by itself not rectify the problem, the number of conformations sampled (1000), the number of conformations re-sampled (three times at 1/10th of the total), and the ensemble docking runs of these conformations are limited by the computational resources available to the authors. The calculations were done on a Linux (Ubuntu 18.04.5 LTS) system with eight cores and a single GPU (GTX 1070). The two computationally expansive steps are generating the ensemble of IDP conformations using the molecular dynamics simulations and the ensemble docking routines required.

The apparent validity of DIBS is based on a broader agreement with the experimental NMR chemical shift changes (Fig. 2a and Fig S2). It demonstrates that EGCG prefers the conformations sampled by the IDP ensemble over the random coils. As noted from the distribution of Rg values (Fig S1c), the IDP conformations show two distinct populations of conformations, a large set (~ 25 Å) and a smaller group (~ 40 Å). In one of the early experimental investigations, Dawson et al. suggest that p53-NTD might function synergistically by combining both unfolded and folded conformations^18^. It is further hypothesized that the p53-NTD domain contains the proline-rich region (PRD residues A63-L93), which tends to adopt a stiff polyproline II (PPII) structure to keep the transactivation domain (TAD1) away from the core domain of the full-length p53^19^. The extended conformations sampled by the MD simulation are consistent with the hypothesis in generating the sub-population of extended conformations. Remarkably, DIBS (and the experimental data) identify only the region close to P70 with a possible binding region in the span of 30 residues of the PRD. A multiscale ensemble modeling of the various areas in the p53-NTD by Terakawa and Takada indicates that including the PRD, the Rg values shift more towards extended conformations^20^. Taken together, it is reasonable to consider that the molecular dynamics of the IDP conformations of p53-NTD reflect the functional diversity of the TAD domains with a second distinct sub-population for conformers due to PRD.

Recently, in the case of NUPR1, a multifunctional IDP similar to the p53-NTD (82 AA), MD simulations typically in the range of 80–200 ns have been utilized^21^. The RMSD plots of the simulations (Fig. S1a) show significantly lesser variability during the time frame of the sampling compared to the starting structures. This means that many intramolecular interactions that occur within the time scale of 500 ns are averaged out. Although molecular dynamics simulations up to 500 ns using an explicit water model could be considered sufficient, a longer time scale, multiple simulations (with a different random seed), and an increase in the repeated sampling would be preferable.

Molecular dynamics trajectories are often clustered to identify sub-groups of structures that share similar conformational properties. However, in an extensive study of ensemble docking of four G-protein-coupled receptors (in membrane environment), Falcon et al. found that ensembles generated from the clustered MD trajectories do not represent the conformations selected by the ligands^9^. These results suggest that repeated independent sampling using random number generation perhaps does not bias the ensembles with pre-selected conformations by clustering.

In a comprehensive perspective, a team of experts led by Smith and co-workers discussed the broader issues related to problems, approaches, and opportunities in conformational sampling and selection of conformations for ensemble docking studies^6^. Although this work, is not providing a solution to what are the 'selectable' conformations, the introduction of control variables such as the random coil ensemble may increase the sensitivity of the search parameters over the free-energy landscape of the apo-protein ensemble by considering a differential free energy selection (between IDP and random coil). If the sampling of conformations used for the ensemble docking between the IDP and random coil ensembles is similar, then EGCG may not differentiate between them. The statistical test suggests that the residues show a significant difference between the data set only on selected amino acid residues of p53-NTD. More importantly, a close match with the experimental results verifies the value of the approach.

DIBS can differentiate IDP ensembles over another (random coil) ensemble via ensemble docking protocols and can offer an alternative approach to conventional results that utilize only the IDP ensemble. The proposed differential probability increases the sensitivity of docking scores as statistical comparison identifies regions of the receptor that may be significantly different from the control set. The DIBS approach presented here would be of value to proteins that are intrinsically disordered or enzymes that have intrinsically disordered regions (IDR). The relatively high computational cost of DIBS compared to standalone docking protocols may be circumvented with scalable molecular dynamics on CPU and GPU architectures. The Eroom's Law (the infamous Moore's law backward) in drug discovery states that the cost for discovering a new drug doubles every nine years^22^. With the increasing computational power towards the availability of exaFLOP machines in the near future, custom-designed systems for MD simulations such as ANTON^23^, as well high throughput simulations coupled with Markov State Models (MSMs)^24^, could make it viable to scale the DIBS. DIBS could be used as a rational screening to select a subset of potential ligands that prefer an ensemble of intrinsically disordered conformations, paving an avenue to the IDP-based drug discovery.

## Methods

### Molecular system

The 93 residues long *N*-terminal domain of the p53 `was obtained from the DISPROT database^25^ in the FASTA format (DP00086r024)^19^. A three-dimensional structure of epigallocatechin gallate (EGCG) was downloaded from the PubChem (CID 65064). The EGCG structure was energy minimized and optimized using the DFT, BYLIP, and a basis-set of 6-31G (d) in the computational program Gaussian^26^.

### Generation of the ensemble of IDP conformations

Ensemble of conformations representative of the intrinsically disordered p53-NTD were generated using molecular dynamics simulations performed using the academic implementation Desmond^27^ combined with the user interface for visualization Schrödinger's Maestro^28^. An extended conformation of the protein was generated within Maestro tools from the primary structure of p53-NTD. The protein-peptide preparation tools with Maestro were used to optimize the starting configuration of the protein structure. The molecular dynamics simulation system was built with an explicit solvent model of water TIP4PD^29^. TIP4PD water model is preferred, as it tends to correct general deficiencies in standard water models, particularly for the disordered proteins in reproducing experimental ensembles. The MD simulations were performed using the default six-step protocol^28^ and at standard conditions of isothermal-isobaric (NPT) ensemble with pressure 1.013 bar (set by Martyna-Tobias-Klein method) and temperature 300 K (Nose–Hoover thermostat). Other simulation parameters are SHAKE algorithm with two fs each for bonded and near interactions and six fs for far interactions with OPLS-AA 2005 force field. Starting from an extended structure built from the primary sequence (FASTA format), MD simulations were performed for 500 ns, and the performance was evaluated using the built-in tools of Desmond. A total of 1000 conformations was collected after the first 100 ns to define the pool of IDP ensembles.

### Generation of random coil ensembles

Starting from the primary sequence of p53-NTD, a representative ensemble of 10,000 random coil structures was generated using the TraDES^30,31^. Then, a subset of 1000 conformations was sampled randomly to define the random coil ensemble of p53-NTD.

### Docking protocol

The molecular docking protocols were performed using the Autodock VINA^32,33^, with the default parameters implemented within YASARA (version 19.1.27) molecular modeling program^34^. The optimized structure of EGCG was imported into YASARA for an ensemble docking protocol using the built-in macro (run_ensemble.mcr). For each ensemble of conformations, the EGCG (ligand) was docked 24 times against each of the 100 p53-NTD (receptor), giving rise to a table consisting of 2400 results ranked according to the binding energy (kcal/mol) and dissociation constant (pM). The results are clustered such that they all differ by at least 5 Å (heavy atom RMSD) along with the amino acid residues involved in the binding process.

The probability score (PS_k_) for each amino acid residue (k) in a particular ensemble run is defined as follows:

1 $$ PS_{k} = \sum\limits_{i} {\frac{1}{N}\left( {{{n_{i} } \mathord{\left/ {\vphantom {{n_{i} } {DC_{i} }}} \right. \kern-\nulldelimiterspace} {DC_{i} }}} \right)} $$

where n_i_ is the number of times the residue (k) encounters the ligand, N total number of the runs (typically number of docking runs × number of structures in the ensemble), and DC_i_ is the corresponding dissociation constant. Thus, Eq. (1) can be considered a weighted sum of the dissociation constant for each amino acid residue in the receptor. The probability factor will be between zero for a residue that does not involve binding events and one whence it is involved in all the binding runs. Each run generated a data set of 2400 (number of docking runs) × 93 (length of p53-NTD), repeated for three independent samplings of sub-structures from the pool of MD generated or random coil structures.

### Statistical analysis

The ensemble docking on the p53-NTD either as an IDP or as random coil sampling generated three sets of probability scores for each. Next, a statistical test was performed between these two data sets to determine which amino acids are responsible for the differential effects between the random coil and the IDP ensembles. Although with three independent runs each, a t-test would be sufficient; a linear model was employed to determine the differential binding score (DIBS), the fold change, and significance (p-values). These statistical methods were based on established protocols and applied to other studies previously^35–37^. Upon completing the statistical test, amino acid residues that are differentially affected between the random coil (control) and the IDP ensembles of p53-NTD are considered significant if fold change (log2) > 2.0 and p-value < 0.05.

### Experimental data from the literature

Experimental NMR data for the p53-NTD have been presented previously by Zhao and Blayney et al.^12^ on the interaction of p53-NTD and EGCG. Chemical shift perturbations of the p53-NTD in the presence of EGCG were measured by following the ^15^N and ^13^CO resonances and were available in the supplementary data provided by the above reference. The experimental data at a p53-NTD: EGCG of 1:4 were used. In addition to using these values directly, the chemical shifts were combined following a Euclidean distance of the measured chemical shifts^38^.

2 $$ \Delta \delta = \sqrt {\frac{1}{2}\left( {\left( {\alpha_{n} \delta_{n} } \right)^{2} + \left( {\alpha_{c} \delta_{c} } \right)^{2} } \right)} $$

where n = ^15^ N, c = ^13^CO, with δ_n_ and δ_c_ are the experimentally observed chemical shift changes due to ligand binding to the protein. Based on the data from BMRB^39^, with the backbone ^15^N, and ^13^CO chemical shift range ~ 22.0 ppm and ~ 32.5 ppm, and defining α_n_ = 22.5/(22.0 + 32.5) = 0.6 and α_c_ = 32.5/(22.0 + 32.5) = 0.4, respectively. Equation (2) was useful as ^15^N and ^13^CO chemical shift changes are opposite due to ligand binding.

All the analyses were performed, and the plots were generated using the R-statistical environment^40^. The protein structural data were analyzed using the tools available in Bio3D^41,42^ and in-house written codes. The IDP or random coil structures' principal component analysis (PCA) represents the maximal variance (percentage of the total mean square displacement of atom positional fluctuations) performed using Bio3D.

## Supplementary Information

Supplementary Figures.
