# Supplementary material for: Identification of ligand binding sites in intrinsically disordered proteins with a differential binding score
Source: Sci Rep. 2021 Nov 19;11:22583. doi: 10.1038/s41598-021-00869-4 (PMC8604960; doi:10.1038/s41598-021-00869-4)
Supplement: Supplementary file 1 — Supplementary Figures. [file 41598_2021_869_MOESM1_ESM.pdf]

Supporting Information

Figure S1: Ensemble generation and characterization of p53-NTD. (a) A plot of the root-mean-squared deviation (RMSD in Å) as a function of the 500 ns MD simulation. (b) A plot of the root-mean-squared fluctuation (RMSF in Å) as the amino acid index function. In panels (a) and (b), Cα, backbone, side-chain, and heavy atoms are shown in different colors. (c) A plot of the distribution of radius of gyration values of the ensembles of MD generated (MD) and random coil (RC) ensembles. (d) Two-dimensional correlation plot between the Rg vs. RMSD for the MD generated ensemble. Panels (e) and (f) principal component analysis (PCA) of the MD generated ensemble (IDP) and the random coils (RC), respectively.

Figure S2: (a) Disorder prediction of p63-NTD. (b) and (c) are the experimental chemical shift data reproduced from the literature (supplemental information from Zhao, J., A. Blayney, et al. [12]).

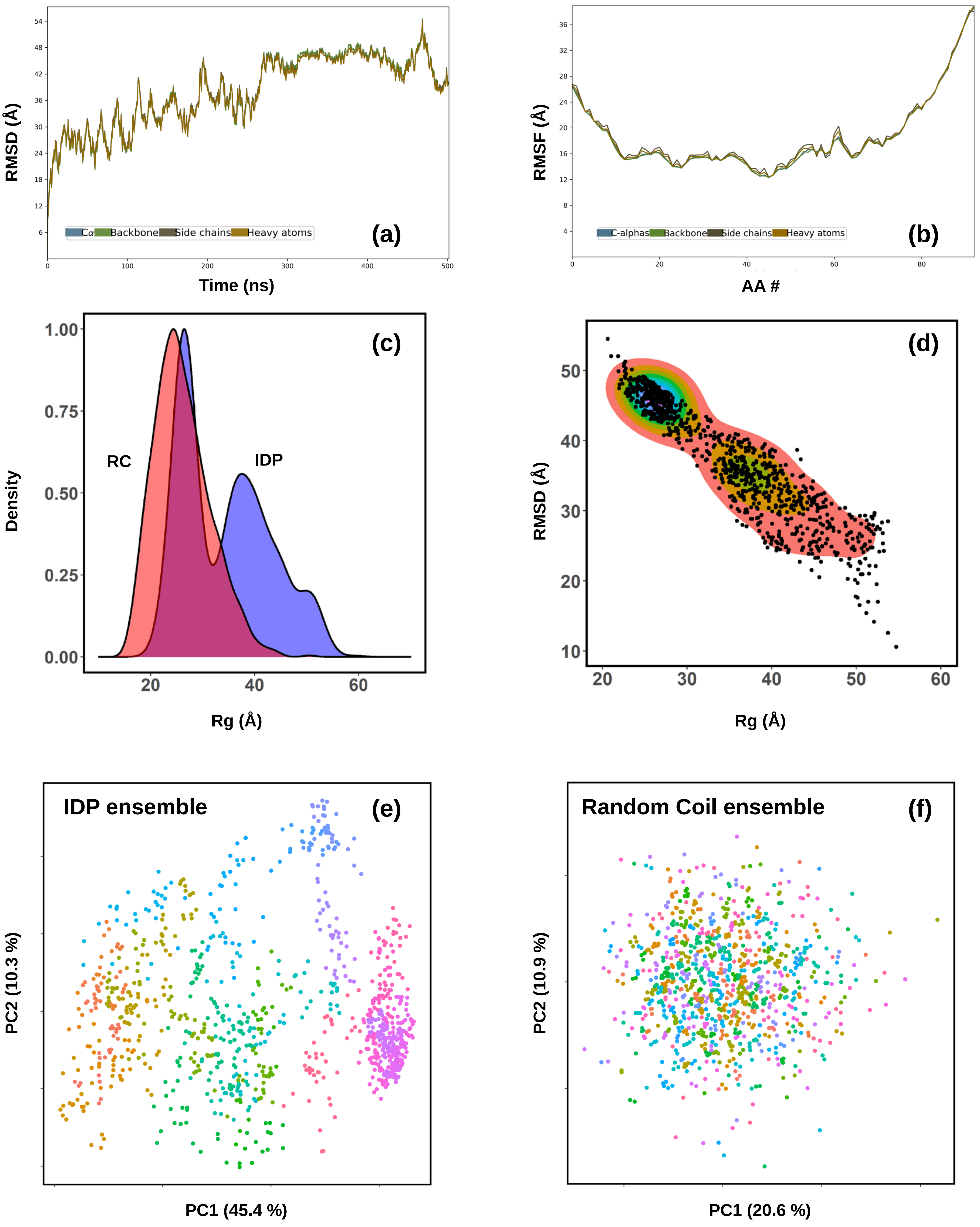

(a)
RMSD (Å)
Time (ns)
Cα Backbone Side chains Heavy atoms
(b)
RMSF (Å)
AA #
C-alphas Backbone Side chains Heavy atoms
(c)
Density
Rg (Å)
RC
IDP
(d)
RMSD (Å)
Rg (Å)
(e)
IDP ensemble
PC2 (10.3 %)
PC1 (45.4 %)
(f)
Random Coil ensemble
PC2 (10.9 %)
PC1 (20.6 %)

Figure S1

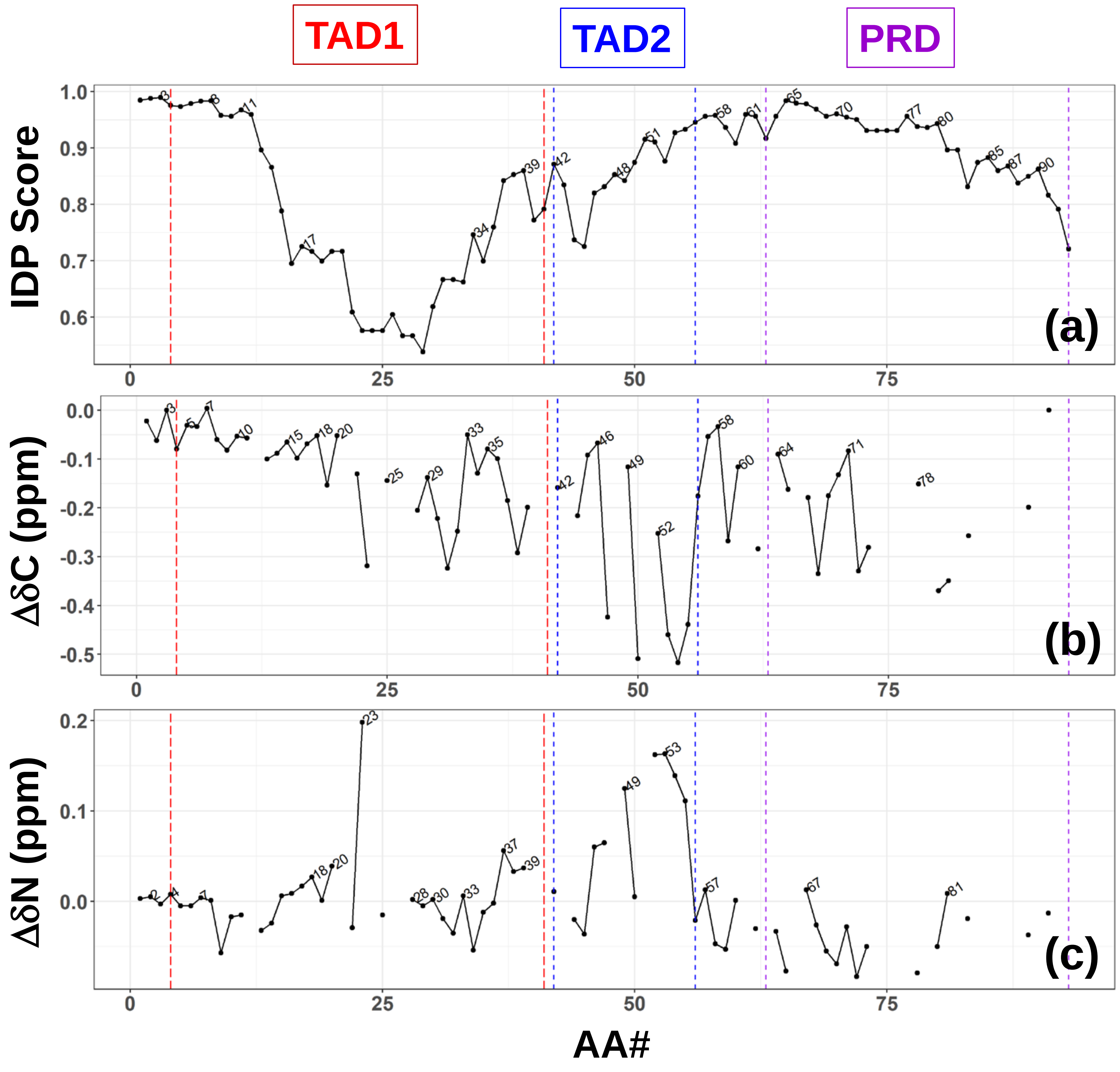

Figure S2
